# Role of MicroRNA 1207-5P and Its Host Gene, the Long Non-Coding RNA *Pvt1*, as Mediators of Extracellular Matrix Accumulation in the Kidney: Implications for Diabetic Nephropathy

**DOI:** 10.1371/journal.pone.0077468

**Published:** 2013-10-25

**Authors:** M. Lucrecia Alvarez, Mahdieh Khosroheidari, Elena Eddy, Jeff Kiefer

**Affiliations:** 1 Diabetes, Cardiovascular, and Metabolic Diseases Center, Translational Genomics Research Institute, Phoenix, Arizona, United States of America; University of Hong Kong, Hong Kong

## Abstract

Diabetic nephropathy is the most common cause of chronic kidney failure and end-stage renal disease in the Western World. One of the major characteristics of this disease is the excessive accumulation of extracellular matrix (ECM) in the kidney glomeruli. While both environmental and genetic determinants are recognized for their role in the development of diabetic nephropathy, epigenetic factors, such as DNA methylation, long non-coding RNAs, and microRNAs, have also recently been found to underlie some of the biological mechanisms, including ECM accumulation, leading to the disease. We previously found that a long non-coding RNA, the plasmacytoma variant translocation 1 (*PVT1*), increases plasminogen activator inhibitor 1 (PAI-1) and transforming growth factor beta 1 (TGF-β1) in mesangial cells, the two main contributors to ECM accumulation in the glomeruli under hyperglycemic conditions, as well as fibronectin 1 (FN1), a major ECM component. Here, we report that miR-1207-5p, a *PVT1*-derived microRNA, is abundantly expressed in kidney cells, and is upregulated by glucose and TGF-β1. We also found that like *PVT1*, miR-1207-5p increases expression of TGF-β1, PAI-1, and FN1 but in a manner that is independent of its host gene. In addition, regulation of miR-1207-5p expression by glucose and TGFβ1 is independent of *PVT1*. These results provide evidence supporting important roles for miR-1207-5p and its host gene in the complex pathogenesis of diabetic nephropathy.

## Introduction

Diabetic nephropathy is a progressive kidney disease that develops consequent to diabetes and is the single leading cause of chronic renal disease worldwide. The disease is characterized by excessive accumulation of extracellular matrix (ECM), with thickening of glomerular and tubular basement membranes and increased production of mesangial matrix, which ultimately progresses to glomerulosclerosis and tubulo-interstitial fibrosis [1]. Despite the tremendous burden in health care costs and the widespread and growing incidence of the disease, there is no effective cure for diabetic nephropathy and the molecular mechanisms underlying its etiology remain only partially understood. Risk factors for diabetic nephropathy are well documented and include duration of diabetes, poor glycemic control, and concomitant presence of hypertension, and/or hyperlipidemia [2], [3]. In addition, genetic factors are also important determinants of disease risk [4], [5], and many variants playing a role in disease susceptibility or development have been identified as a result of both candidate gene investigations and genome-wide association studies [4], [6]. Recent evidence has also shown involvement of epigenetic factors, such as DNA methylation, histone post-translational modifications, microRNAs (miRNAs), and long non-coding RNAs, in modulating the development of renal diseases, including diabetic nephropathy [5]. Of these epigenetic factors, the vast majority of research has focused on miRNAs, several of which, including miR-192, miR-216a, miR-217; miR-377, miR-21 and miR-29c, appear to play a substantial role in biological mechanisms leading to the development of diabetic nephropathy [7], [8].

We previously reported association of variants in the gene encoding plasmacytoma variant translocation 1 (*PVT1*) with ESRD attributed to type 2 diabetes (T2D) [9], and subsequently validated this locus in a replication study comprised of individuals with ESRD attributed to type 1 diabetes (T1D) [10]. We also found *PVT1* to be expressed in a variety of renal cell types, although the role of the gene in the kidney is not known [10].

Long non-coding RNAs (lncRNAs) are transcribed RNA molecules (>200 nt in length) that structurally resemble mRNAs but do not encode proteins. *PVT1* is classified as a lncRNA because it is a long RNA (1.9 kb) that encodes a number of alternative transcripts but does not produce a protein. In addition, *PVT1* is classified as a non-coding RNA (NR_003367.2) in the NCBI website (http://www.ncbi.nlm.nih.gov/nuccore/NR_003367.2). *PVT1* is the first long non-coding RNA that has been associated with kidney disease. Recently, we identified possible molecular mechanisms by which this non-coding RNA may contribute to the development and progression of diabetic kidney disease [11]. We found that *PVT1* knockdown in human mesangial cells significantly reduced mRNA and protein levels of the major ECM proteins, fibronectin and collagen, type IV, alpha 1, and two key regulators of ECM proteins, transforming growth factor beta 1 (TGF-β1) and plasminogen activator inhibitor 1 (PAI-1) [12]–[14]. Based on these findings, we hypothesized that *PVT1* may contribute to the increased accumulation of ECM in the glomeruli characteristic of diabetic nephropathy. However, *PVT1* is known to give rise to at least six miRNAs, including miR-1204, -1205, -1206, -1207-5p, 1207-3p, and -1208 [15], and we could not discount the possibility that the glucose-mediated effects of *PVT1* on ECM accumulation were due to one or more of the *PVT1*-derived miRNAs, instead of, or in addition to, the long non-coding *PVT1* transcript itself. The goal of this study, therefore, was to investigate both the independent and combined effects of *PVT1* and its miRNAs on ECM accumulation. Here we show that miR-1207-5p is the major *PVT1*-derived miRNA in renal cells. We also determine that levels of both miR-1207-5p and its host gene *PVT1* increased in response to glucose; however, expression of these two genes is independent of one another. Both miR-1207-5p and *PVT1* together, but independently, increase expression of TGF-β1 and PAI-1, the two key regulators of ECM accumulation in the glomeruli. These results provide evidence supporting important roles for miR-1207-5p and its host gene in the pathological processes underlying the development of diabetic nephropathy.

## Results

### Quantification of PVT1-derived microRNAs in different types of kidney cells

**Figure 1:**
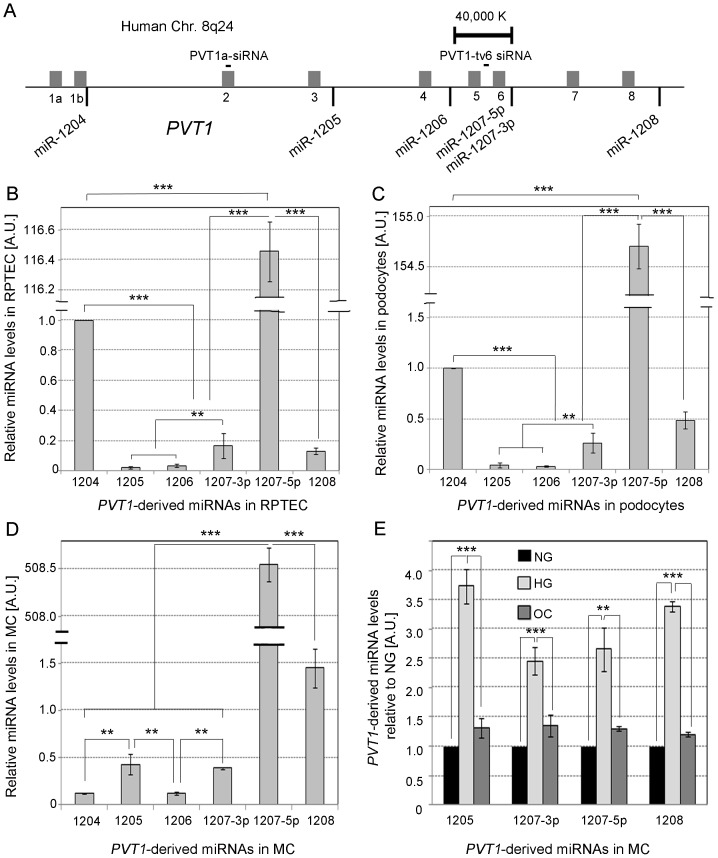
*PVT1*-derived miRNAs. A. Location of the validated *PVT1*-derived miRNAs. The grey bars represent the nine *PVT1* exons. Panels **B**, **C** and **D**, relative quantification of *PVT1*-derived miRNAs in human renal proximal tubule epithelial cells (RPTEC), podocytes, and mesangial cells (MC) by TaqMan qPCR, respectively. RNU6B and RNU44 were used as endogenous controls. **E**. Relative quantification of miRNAs in MC grown for 48 h in MsBM medium supplemented with 5% FBS, containing either normal glucose (NG: 5.6 mM), NG +3-O-methyl-D-glucose (3-O-MG) to control for osmotic effects (OC: 5.6 mM glucose +24.4 mM 3-O-MG), or high glucose (HG: 30 mM). Results represent average of three independent experiments. Data are means ± S.D. A.U.: arbitrary units. The significance is indicated only for samples that are significant different from all the others. * *P*<.05; ** *P*<0.01; *** *P*<0.001.

The locations of the six miRNAs mapping to the *PVT1* locus, including miR-1204, -1205, -1206, -1207-5p, 1207-3p, and -1208 [15] are shown in Fig. 1A. Both miR-1207-3p and miR-1207-5p are separately encoded inside the PVT1 gene and derived from a common precursor. We first investigated the relative level of expression of these six miRNAs in normal human renal proximal tubule epithelial cells (RPTEC, Fig. 1B), podocytes (Fig. 1C), and normal human mesangial cells (MC). We found that levels of miR-1207-5p were at least 100X higher compared to the other *PVT1*-derived miRNAs in all three types of kidney cells (Fig. 1D). We next evaluated the effect of glucose on levels of the four *PVT1*-derived miRNAs expressed most abundantly in MC: miR-1205, miR-1207-3p, miR-1207-5p and miR-1208. As shown in Fig. 1E, glucose treatment upregulated expression of miR-1207-3p and miR-1207-5p by 2.5 fold and miR-1205 and miR-1208 by 3.5 fold.

### Comparison of PVT1 and miR-1207-5p levels in different human tissues and kidney cell types

**Figure 2:**
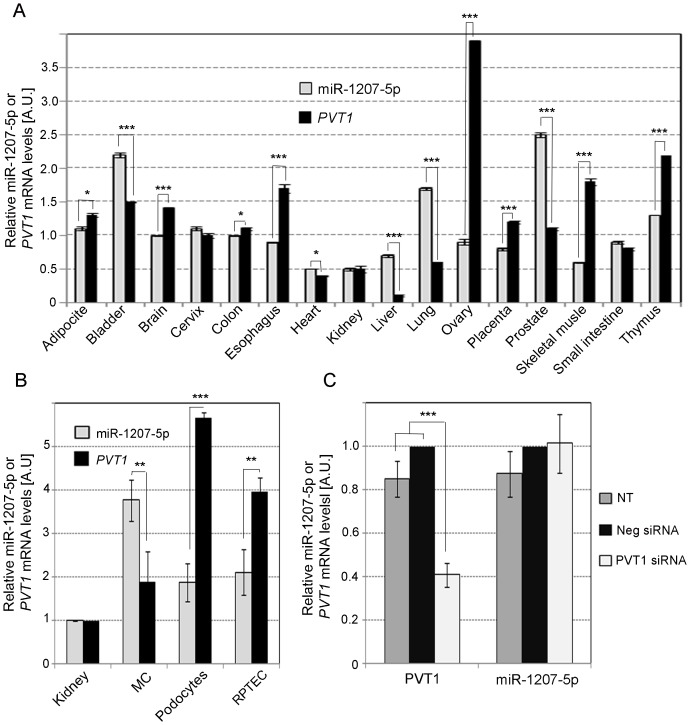
Comparison of *PVT1* and miR-1207-5p expression. A. Expression of miR-1207-5p and *PVT1* in different normal human tissues using the Human Total RNA Survey Panel (Life Technologies). Relative quantification of miR-1207-5p and *PVT1* mRNA was performed by TaqMan qPCR. Results represent average of three quantifications of the same sample and each sample contains tissues from three different donors. Data are means ± S.E.M. **B**. Relative quantification of miR-1207-5p and *PVT1* in whole kidney and different types of kidney cells by TaqMan qPCR. **C**. Levels of *PVT1* mRNA and miR-1207-5p in MC transfected with 30 nM of PVT1 siRNA or negative control (Neg) siRNA. RNU6B, RNU44, UBC and PPIA were used as endogenous controls (see Table S2). In panels B and C, results represent averages from three independent experiments, and data are means ± SD. NT: no treatment. The significance is indicated only for samples that are significant different from all the others. * *P*<0.05; ** *P*<0.01; *** *P*<0.001.

Because miR-1207-5p is expressed at much higher levels than the remaining *PVT1*-derived miRNAs (Fig. 1B-D), we focused the remainder of our experiments on this molecule. We first sought to determine whether expression of miR-1207-5p and *PVT1* is interrelated using a range of different normal human tissues. As shown in Fig. 2A, expression of miR-1207-5p and *PVT1* was not correlated at levels that reached statistical significance (Spearman r =  0.27; *P* = 0.3119). In comparisons of expression in MC, RPTEC, and podocytes (Fig. 2B), levels of miR-1207-5p and *PVT1* were again not significantly correlated (Pearson r =  −0.9399; *P* =  0.2219). To further investigate the relationship between the two genes, we knocked down *PVT1* expression in MC and measured levels of both *PVT1* and miR-1207-5p. We observed that levels of *PVT1* mRNA were reduced 60%, but expression of miR-1207-5p remained unchanged (Fig. 2C).

### TGF-β1 dose-response and time-course effect on miR-1207-5p

**Figure 3:**
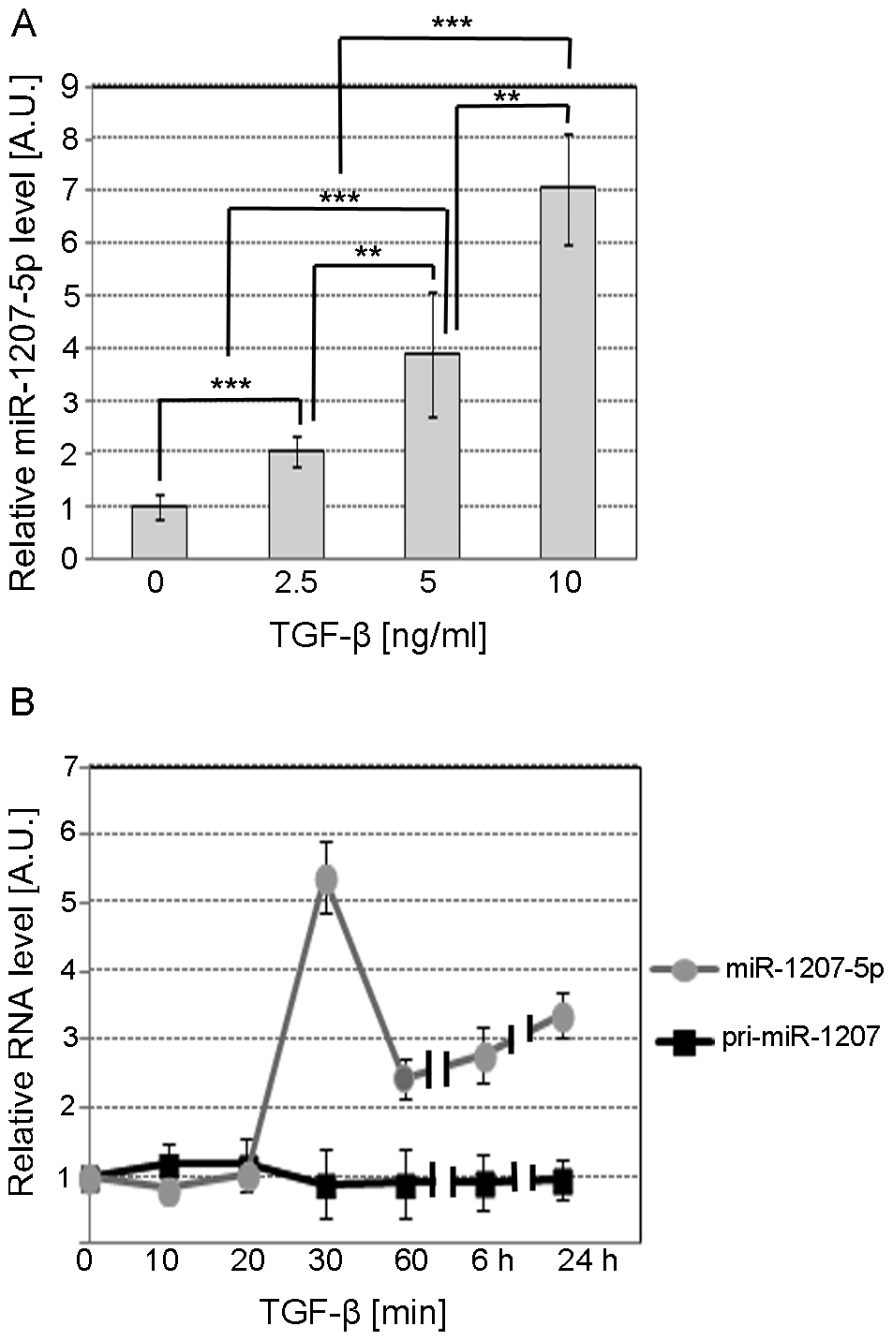
TGF-β1 dose-response and time-course effect on miR-1207-5p. **A**. TaqMan qPCR relative quantification of miR-1207-5p in MC treated for 24 h with serum-free medium supplemented with 2.5, 5.0, or 10.0 ng/ml TGF-β1. **B**. Relative quantification of miR-1207-5p and pri-miR-1207 in MC treated for different times with serum-free medium supplemented with 10 ng/ml TGF-β1. RNU6B, UBC, and 18S RNA were used as endogenous controls. Results represent averages from three independent experiments. Data are means ± SD. A.U.: arbitrary units. The significance is indicated only for samples that are significant different from all the others. * *P*<0.05; ** *P*<0.01; *** *P*<0.001.

It is well known that primary miRNAs (pri-miRNAs) containing a Smad-binding sequence in the stem region are processed less efficiently by Drosha when Smad proteins are not available [16]-[18]. However, when Smad proteins migrate from the cytoplasm to the nucleus in response to TGF-β, they bind to their cognate binding site in pri-miRNAs leading to Drosha recruitment and enhanced miRNA processing [18]. Because pri-miR-1207-5p contains a Smad-binding sequence in the stem region, we sought to assess the effect of TGF-β1 on miR-1207-5p expression in a dose- and time-dependent manner. As shown in Fig. 3A, we observed a strong TGF-β1 dose-response effect on levels of mature miR-1207-5p. We also found a biphasic effect of TGF-β1 on miR-1207-5p expression over time, with an initial 5-fold increase at 30 min, followed by a second, slower rise from 2.4-fold at 6 h to 3.3-fold at 24 h incubation with TGF-β1 (Fig. 3B).

### Validation of putative miR-1207-5p target genes G6PD, PMEPA1, PDPK1, and SMAD7

**Figure 4:**
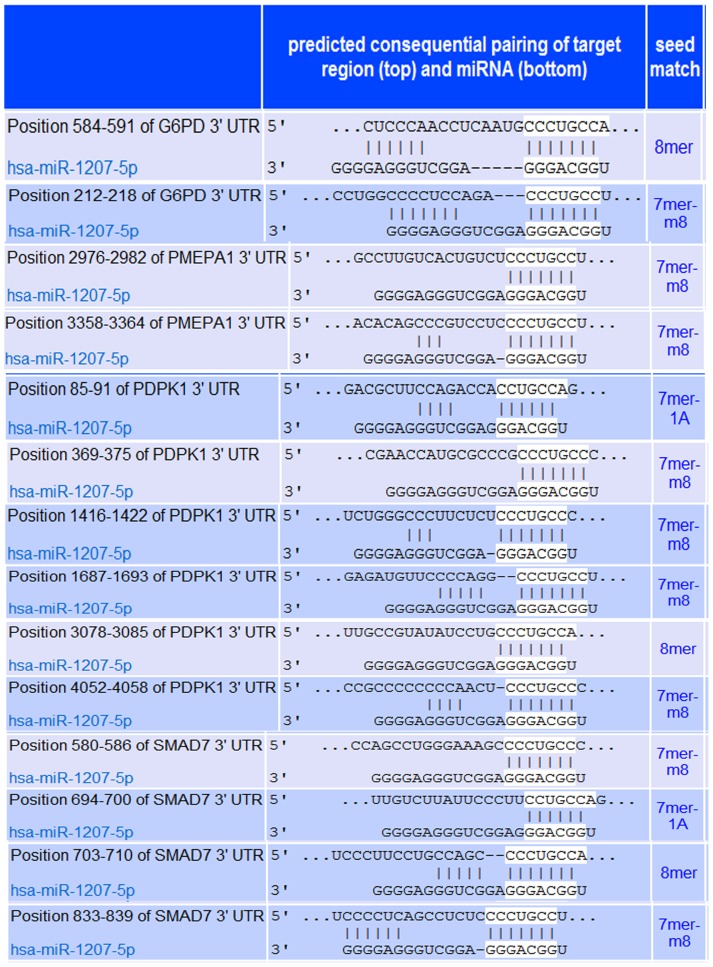
Predicted pairing of target region (top) and miR-1207-5p (bottom) according to TargetScan (www.targetscan.org). A total of 14 miR-1207-5p's target sites are predicted in the four target genes selected: *G6PD, PMEPA1, PDPK1, and SMAD7. G6PD*, glucose-6-phosphate dehydrogenase; *PMEPA1*, prostate transmembrane protein, androgen induced 1; *PDPK1*, 3-phosphoinositide dependent protein kinase-1; *SMAD7*, SMAD family member 7.

**Figure 5:**
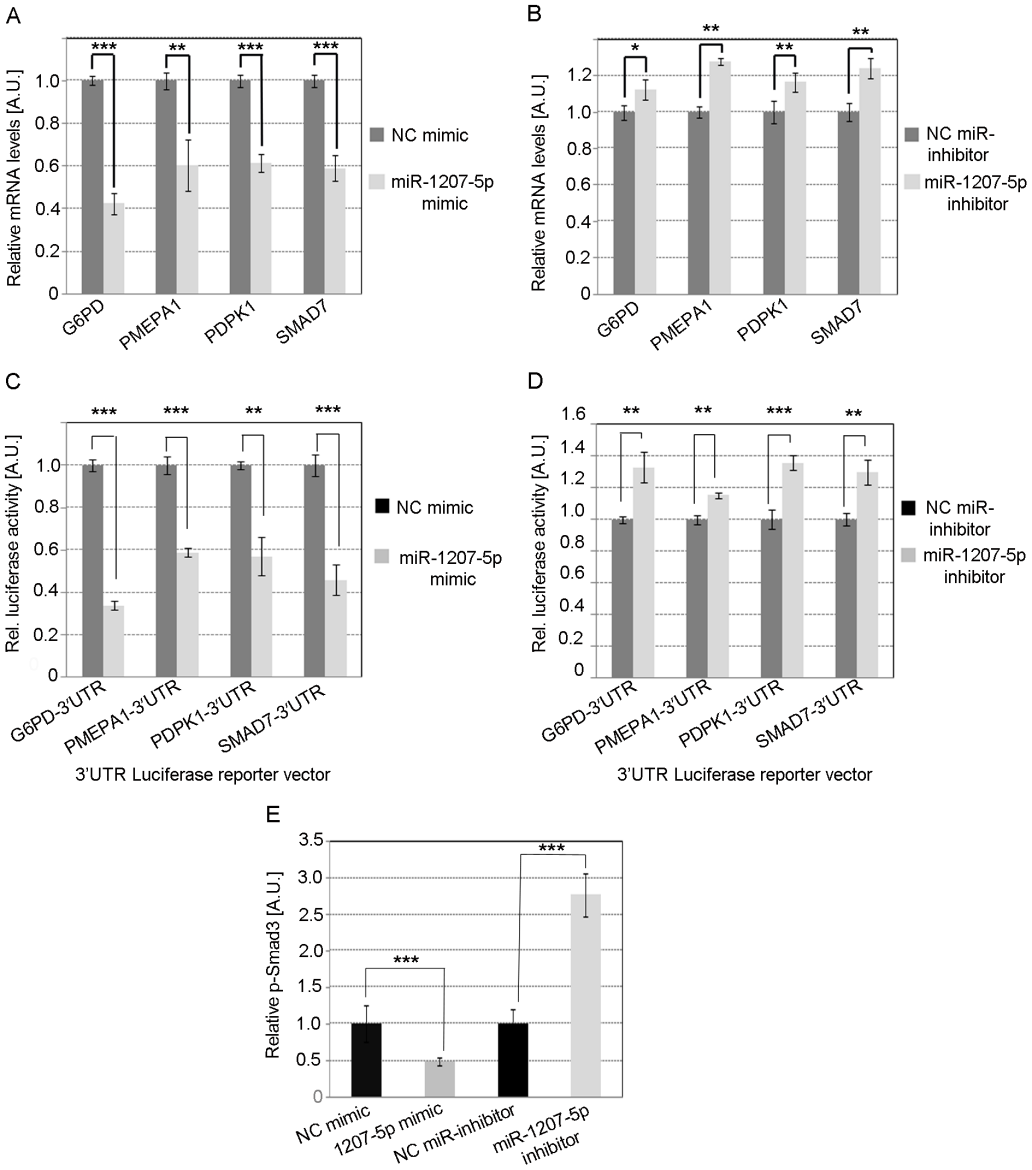
Validation of putative miR-1207-5p target genes. miR-1207-5p target genes were identified by TargetScan software and four genes were selected using Pathway Studio software based on potential biological effects relevant to ECM accumulation. The four genes selected, *G6PD, PMEPA1, PDPK1, and SMAD7*, were validated as putative miR-1207-5p target genes in MC using 25 nM miR-1207-5p or NC mimic (**A**), and 50 nM anti-miR-1207-5p or negative control (**B**). Results in A and B represent averages from three independent experiments. Data are means ± SD. About 20,000 human embryonic kidney 293 (HEK293) cells were seeded per well into a white 96-well plate and co-transfected with 100 ng of the indicated 3′UTR luciferase reporter vectors and 30 nM miR-1207-5p or negative control mimic (Dharmacon) using 0.2 µl per well of Lipofectamine 2000 (**C**). HEK293 cells were also co-transfected with reporter vectors and 50 nM miR-1207-5p inhibitor or negative control (Dharmacon) (**D**). Luciferase activity was measured 48 h after transfection using the Dual-Glo Luciferase Assay System (Promega). Firefly luciferase activity was normalized to the corresponding renilla luciferase activity and plotted as a percentage of the control (HEK293 cells co-transfected with plasmid and control mimic or inhibitor). Approximately 70,000 MC/well were seeded in a 12-well plate and transfected with 3 µl Lipofectamine RNAiMAX (Life Technologies) mixed with 30 nM miR-1207-5p or negative control mimic, and 50 nM anti-miR-1207-5p or negative control. About 24 h after transfection, cells were stimulated with high glucose [30 mM] and incubated for another 24 h. Afterwards, cell culture media was removed, and cells were lysed and phosphorylathed Smad3 was quantified using phospho-SMAD3 (ser423/425) Instant One ELISA (eBiosciences), as per manufacturer's instructions (**E**). Experiments showed in C, D, and E were performed in quadruplicate. The significance is indicated only for samples that are significant different from all the others. * *P*<0.05; ** *P*<0.01; *** *P*<0.001. A.U.: arbitrary units. *G6PD*, glucose-6-phosphate dehydrogenase; *PMEPA1*, prostate transmembrane protein, androgen induced 1; *PDPK1*, 3-phosphoinositide dependent protein kinase-1; *SMAD7*, SMAD family member 7; TGF-β, transforming growth factor beta; PAI-1, plasminogen-activator inhibitor 1; MC, mesangial cells; NC, negative control; p-Smad3, phosphorylated Smad3.

Our next step was to identify downstream effects on potential miR-1207-5p targets. We first identified potential target genes using TargetScan [19]–[21] and Pathway Studio software [22], and then selected four of them based upon biological function for validation using miRNA mimics and inhibitors: *G6PD* (glucose-6-phosphate dehydrogenase), *PMEPA1* (prostate transmembrane protein, androgen induced 1), *PDPK1* (3-phosphoinositide dependent protein kinase-1), and *SMAD7* (SMAD family member 7) (Fig. 4 and Table 1). We found that treatment with miR-1207-5p mimic significantly decreased levels of *G6PD, PMEPA1, PDPK1, and SMAD7* mRNA compared to control (Fig. 5A), while inhibition of miR-1207-5p increased expression of all target genes (Fig. 5B).

**Table 1:** Analysis of ECM accumulation-related miR-1207-5p targets using TargetScan and miRanda.

		TargetScan^1^							miRanda^2^
			Conserved sites^3^			Poorly conserved sites			
Gene symbol	Gene name	Context score^4^	n	Type^4^	Target position (3′UTR region)	n	Type^5^	Target position (3′UTR region)	mirSVR score
*G6PD*	Glucose-6-phosphate dehydrogenase	−0.71	1	8mer	584–591	1	7mer-m8	212–218	−0.91
*PMEPA1*	Prostate transmembrane protein, androgen induced 1	−0.34	0			2	7mer-m8	2976–2982; 3358–3364	−0.12
*PDPK1*	3-phosphoinositide dependent protein kinase-1	−0.42	0			6	8mer, 7mer-m8, 7mer-1A	85–91, 369–375, 1416–1422, 1687–1693, 3078–3085, 4052–4058	−0.13
*SMAD7*	SMAD family member 7	−0.55	0			4	8mer, 7mer-m8, 7mer-1A	580–586, 694–700, 703–710, 833–839	−0.42

1 TargetScan website: http://www.targetscan.org.

2 miRanda website: http://www.microrna.org.

3 Conserved across most mammals.

4 Predicted efficacy of targeting [20].

5 Type of site: TargetScan predicts biological targets of miRNAs by searching for the presence of conserved 8mer and 7mer sites that match the seed region of each miRNA. **7mer-1A sites**: exact match to positions 2–7 of the mature miRNA (the seed) followed by an “A” (adenine). **7mer-m8 sites**: exact match to positions 2–8 of the mature miRNA (the seed + position 8). **8mer** **sites**: exact match to positions 2–8 of the mature miRNA followed by an “A” (adenine). 7mer-m8 sites are defined [19].

Next, we sought to determine whether the effect of miR-1207-5p on the expression of *G6PD, PMEPA1, PDPK1, and SMAD7* was a consequence of the direct binding of miR-1207-5p to the 3′ untranslated (UTR) region of these genes. To this end, we constructed four luciferase reporter plasmids containing the 3′UTR of *G6PD, PMEPA1, PDPK1, and SMAD7* cloned into the pmirGLO Dual Luciferase miRNA Target Expression Vector (Promega; Fitchburg, WI) to generate four reporter plasmids: pG6PD-3′UTR, pPMEPA1-3′UTR, pPDK1-3′UTR, and pSMAD7-3′UTR. We found that luciferase activity decreased in HEK293 cells co-transfected with each of the four reporter plasmids and miR-1207-5p mimics (Fig. 5C), and increased when miR-1207-5p was knocked down (Fig. 5D), suggesting that *G6PD, PMEPA1, PDPK1*, and *SMAD7* are direct targets of miR-1207-5p. Because three of these target genes (*PMEPA1, PDPK1*, and *SMAD7*) block the activation of TGF-β/Smad (see Discussion section), we sought to confirm the indirect effect of miR-1207-5p on the levels of activated Smad3 (p-Smad3). We found that phosphorylated Smad3 (p-Smad3 or activated Smad3) increased in MC cells overexpressing miR-1207-5p while decreased in cells with miR-1207-5p knockdown compared to controls (Fig. 5E).

### Effects of PVT1 and miR-1207-5p on ECM accumulation

**Figure 6:**
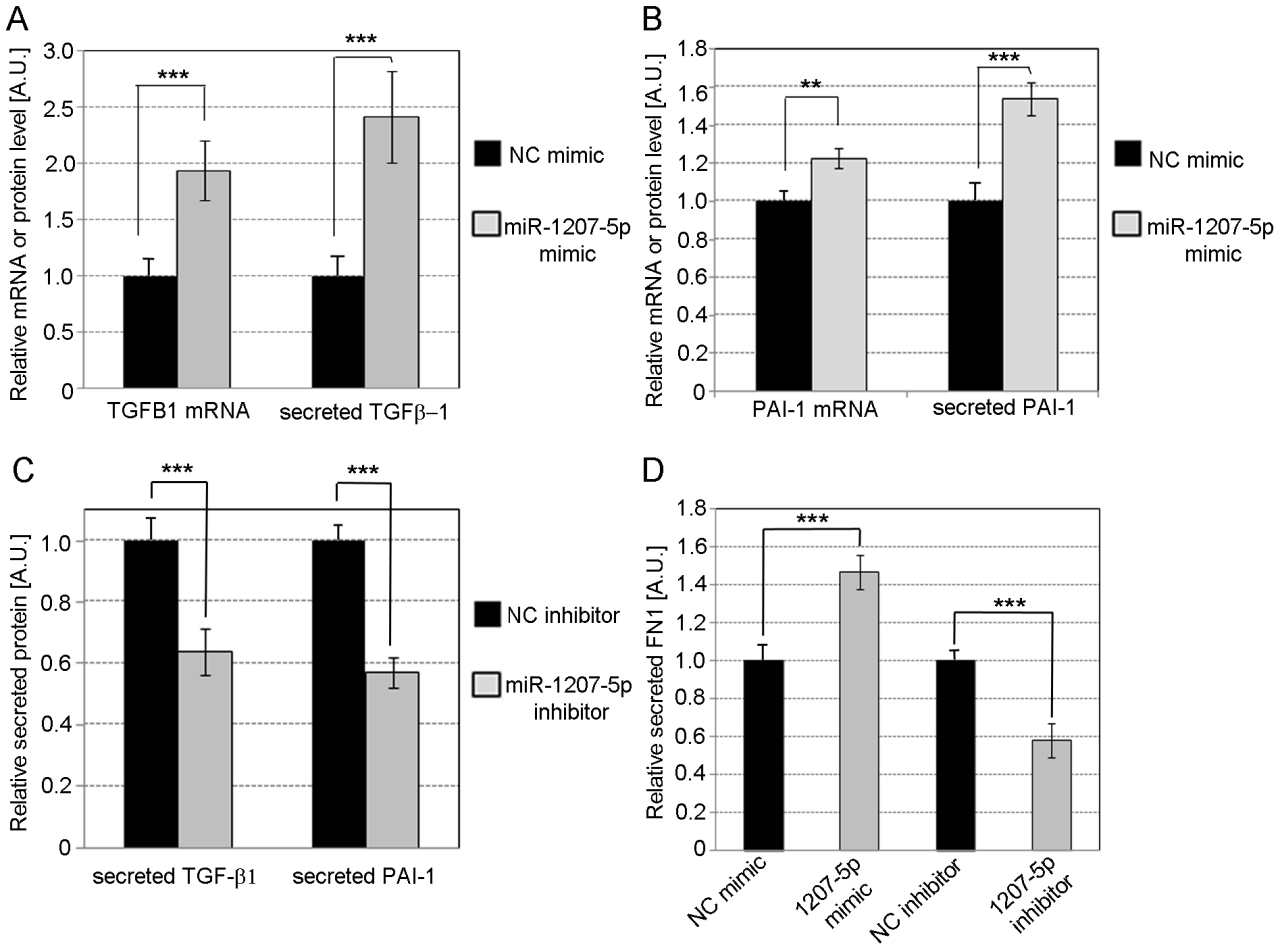
Effect of miR-1207-5p on TGF-β1, PAI-1 and FN1. Relative quantification of TGF-β1 (**A**) and PAI-1 (**B**) mRNA and secreted protein in MC over-expressing miR-1207-5p compared to control. (**C**) Relative TGF-β1 and PAI-1 protein secreted by MC transfected with miR-1207-5p inhibitor or NC inhibitor compared to control. (**D**) Relative quantification of secreted FN1 from MC with miR-1207-5p over-expression or knockdown compared to control. Cells were transfected with 3 µl Lipofectamine RNAiMAX mixed with 30 nM miR-1207-5p or negative control (NC) mimic, or 50 nM of miR-1207-5p inhibitor or NC inhibitor. Specific mRNAs were quantified by TaqMan qPCR using PPIA and UBC as endogenous controls. Secreted TGF-β1, PAI-1, and FN1 were determined by ELISA. Results represent averages from three independent experiments. Data are means ± SD. A.U.: arbitrary units. The significance is indicated only for samples that are significant different from all the others. * *P*<0.05; ** *P*<0.01; *** *P*<0.001.

Because diabetic nephropathy is characterized by excessive accumulation of ECM in the glomeruli, we next investigated the effect of miR-1207-5p and *PVT1* over-expression and knockdown on the level of the two main regulators of ECM proteins, TGF-β1 and PAI-1 as well as one of the major components of ECM in MC: FN1. In MC transfected with miR-1207-5p mimics, we observed increases in mRNA and protein levels of TGF- β1 (Fig. 6A) and PAI-1 (Fig. 6B) compared to controls. In contrast, we found a decrease in secreted TGF-β1 and PAI-1 after miR-1207-5p knockdown compared to controls (Fig. 6C). In addition, we observed a 40% increase or decrease compared to controls in the levels of secreted FN1 in MC transfected with miR-1207-5p mimics or inhibitors, respectively (Fig. 6D).

**Figure 7:**
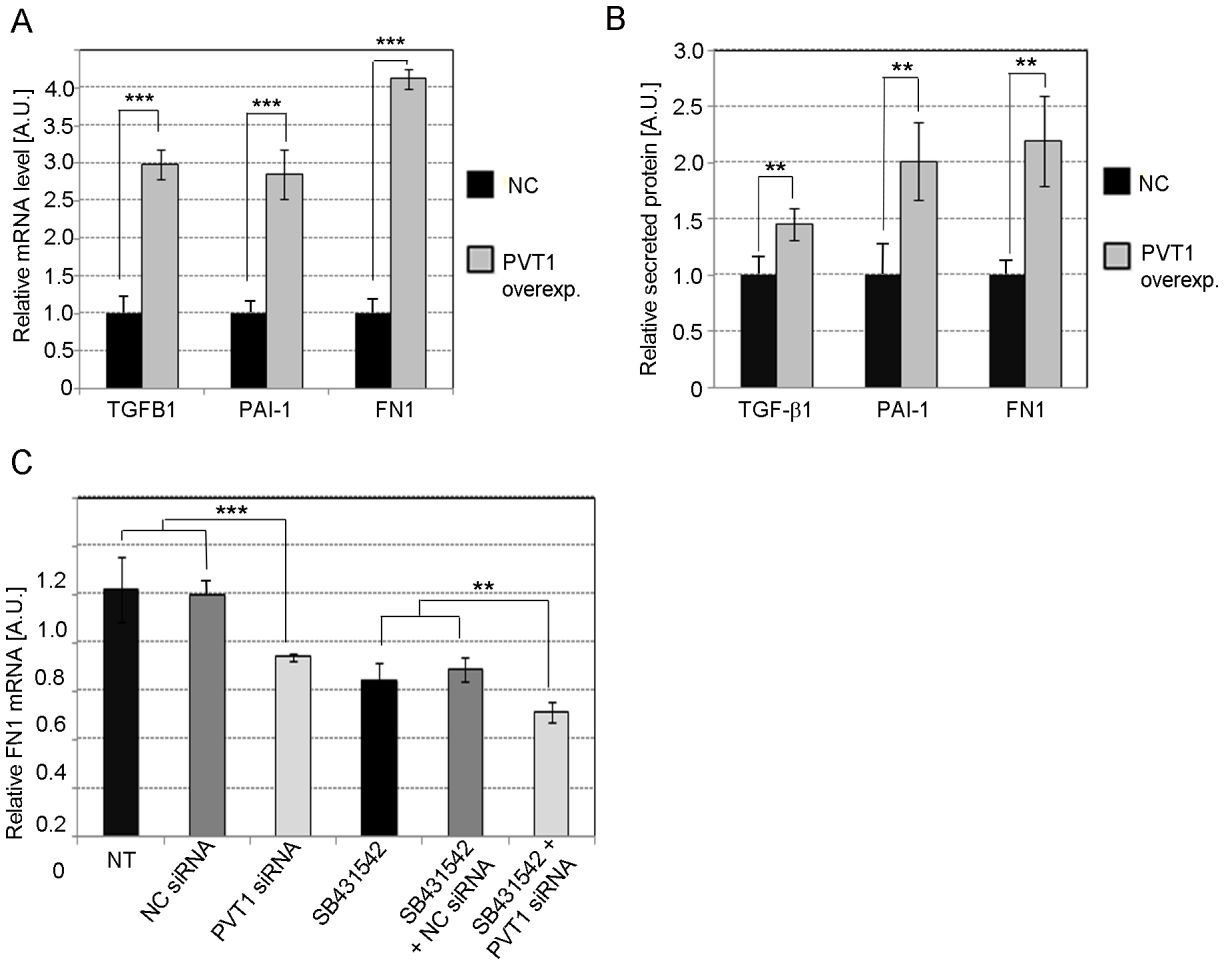
Effect of *PVT1* on TGF-β1, PAI-1 and FN1. Relative quantification of TGF-β1, PAI-1, and FN1 mRNA (**A**) or secreted protein (**B**) in MC with *PVT1* over-expression. Cells were transfected with plasmid pCMV-PVT1 or empty vector pCMV (NC, negative control) by electroporation using the Neon System. (**C**) Changes in expression of FN1 mRNA in MC treated with 30 nM *PVT1* or NC siRNA, in the presence or absence of the TGF-β signaling inhibitor SB431542. NC siRNA comprised of sequence not found in the human genome. MC were transfected using 5 µl Lipofectamine RNAiMax (Life Technologies) per ml of MsBM media. Results represent averages from three independent experiments. Data are means ± SD. A.U.: arbitrary units. The significance is indicated only for samples that are significantly different from all the others. * *P*<0.05; ** *P*<0.01; *** *P*<0.001.

Similarly, in MC with *PVT1* over-expression, levels of TGFB1, PAI-1, and FN1 mRNA (Fig. 7A) or secreted protein (Fig. 7B) significantly increased compared to the negative control (NC) or empty vector, confirming our previous results following *PVT1* knockdown [11]. To determine whether the effect of *PVT1* on the expression of FN1 was dependent on TGF-β1, we transfected MC with 30 nM *PVT1* siRNA or NC siRNA in the presence or absence of 1 µM SB431542, a potent and specific inhibitor of TGF-β type I receptor that specifically inhibits TGF-β signaling but has no effect on BMP signaling [23]. We observed a 30% decrease in the level of FN1 mRNA in cells treated with SB431542 and NC mimic, and a further 20% decrease when cells were incubated with both SB431542 and *PVT1* siRNA (Fig. 7C).

## Discussion

We have previously shown that variants in *PVT1* are associated with diabetic kidney disease [9], [10], and that its expression is strongly regulated by glucose [11]. *PVT1* also regulates the expression of ECM-related proteins [11], and combined, these results suggest a role for this gene in the development of diabetic nephropathy. *PVT1* is a long non-coding RNA (lncRNA), which is a transcribed RNA molecule (>200 nt in length) that structurally resemble mRNAs but does not encode proteins [24]. LncRNAs contain individual domains that allow them to specifically associate with DNA, RNA, and/or protein [25]. Some mammalian lncRNAs have a role in post-transcriptional regulation of gene expression by binding to miRNAs, and consequently preventing specific miRNAs from binding to their target mRNAs [26].

*PVT1* is the first and, so far, the only, lncRNA that has been associated with kidney disease. However, it is not known whether the glucose-induced effects of *PVT1* on ECM accumulation are due to the *PVT1* transcript itself or any of the six miRNAs mapping to the locus.

In the current study, we sought to investigate the relationship between *PVT1* and *PVT1*-derived miRNAs in ECM accumulation. We found that miR-1207-5p is the most abundantly expressed *PVT1*-derived miRNA in a number of different types of renal cells. However, expression of the intronic miR-1207-5p and its host gene, *PVT1*, is not significantly correlated in different types of normal human tissues and kidney cells, suggesting that expression of one is not dependent on that of the other. Nevertheless, in some tissues may exist a common upstream regulation that makes miR-1207-5p and *PVT1* levels change together in the same direction. Although most of the intronic miRNAs are co-regulated with their host genes, there are many examples in which the expression patterns of intronic miRNAs are different from the host RNAs [27]–[29]. Liang et al. [27] found that among 31 intronic miRNAs quantified, 7 of them (22%) had no significant correlation with their host genes in expression among 19 tissue types. In addition, Monteys et al. [30] reported that ∼35% of intronic miRNAs can be transcribed from Pol II or Pol III intron-resident promoters, independent of the host gene promoter.

Barsotti et al. [31] recently demonstrated a p53-dependent induction of endogenous *PVT1* transcripts and consequent up-regulation of one of its encoded miRNAs, miR-1204. These authors found a p53 binding site between *PVT1* exon 1A and 1B (see Fig. 1A), close to miR-1204. Fig 1A shows that miR-1204 is the *PVT1*-encoded miRNAs that is closest to the *PVT1* promoter and, therefore, it is likely that miR-1204 does not have its own promoter but depends on the host's promoter for its transcription. Thus, it is expected that the induction of *PVT1* by p53 up-regulates both *PVT1* and subsequently miR-1204. According to Barsotti et al. [31], p53 also up-regulates pri-miR-1207 but since miR-1207-5p is located far from *PVT1* promoter (Fig. 1A), it is unlikely that the observed increase is just a consequence of the increased *PVT1* transcription. Thus, we speculate that another p53 binding site/s located in the promoter region of pri-miR1207 might be inducing its transcription independently from *PVT1*.

The tumor suppressor p53 has been implicated in the transcriptional regulation of profibrotic effectors. For example, p53 binds to motifs in the PAI-1 promoter and induces its expression. It is believe that phosphorylated p53 and SMAD2 form a transcriptionally active multi-protein complexes involved in a subset of TGF-β1 responses such as PAI-1 gene control. Regardless of the precise mechanism, p53 has a central role in TGF-β1-initiated PAI-1 gene control [32]. It is known that p53 induces renal fibrosis, podocyte apoptosis, insulin resistance, and promotes different kidney diseases [33]–[35]. A recent report from Deshpande et al. [33] suggests that p53 and miR-192 up-regulates each other's expression under diabetic conditions, and might play a role in the pathogenesis of diabetic nephropathy by inducing hypertrophy and fibrosis of glomerular mesangial cells. Similarly, we speculate that p53 might activate *PVT1* and miR-1207 promoters under diabetic conditions resulting in renal fibrosis. In addition, p53 might enhance the post-transcriptional maturation of miR-1207 [36] contributing to increased levels of this miRNA and subsequent fibrosis.

We sought to determine if *PVT1* knockdown would affect the levels of the intronic miR-1207-5p. Although the mechanism of siRNA-mediated RNAi involves targeting mature mRNA (without introns) for cleavage and destruction in the cytoplasm, there are evidences that human RISCs programmed with siRNA are also present in the nucleus and can knock down primary RNA transcripts, which contain introns [37]–[39]. Thus, we found that in spite of a 60% decrease in *PVT1* mRNA in MC following *PVT1* knockdown, miR-1207-5p remained unchanged. Therefore, the effect of *PVT1* on the accumulation of ECM in MC cultured under hyperglycemic conditions that we previously described [11], is independent of miR-1207-5p. This phenomenon has been observed in earlier studies, in which two miRNAs that contribute to diabetic nephropathy, miR-216 and miR-217, are both located in an intron of a long non-coding RNA RP23-298H6.1-0001 [40]. However, in contrast with our results, the expression of miR-216 and miR-217 appears to be dependent of the host gene *RP23*, which is regulated by TGF-β and miR-192 through E-boxes present in its promoter. Apparently, the sole function of *RP23* is to generate miR-216 and miR-217, and no other effects independent from the miRNAs encoded within its locus are known, unlike the relationship between *PVT1* and miR-1207-5p showed here. In addition, the same group recently reported that miR-192, another miRNA associate with diabetic nephropathy, is co-regulated with its potential host gene, the non-coding RNA CJ241444, which is controlled by TGF-β through Smad binding elements (SBEs) in the promoter [41]. Here, we hypothesize that the expression of miR-1207-5p is not co-regulated with *PVT1* because this miRNA has its own promoter, which makes it independent from the expression of the host. In most of intronic miRNAs, the primary transcripts are cleaved by Drosha from the host RNA to release ∼70 nt pre-miRNAs that are subsequently processed by Dicer to generate mature ∼22 nt miRNAs [42]. However, other intronics miRNAs, particularly those located far from the host promoter such as miR-1207-5p, may have their own promoters and, therefore, their expression and regulation maybe be independent of their host gene [30].

We found that miR-1207-5p is up-regulated by TGF-β1 in MC in a dose- and time-dependent manner. Despite an increase in the level of the mature miRNA, expression of the primary transcript product from the miR-1207 gene, pri-miR-1207, did not change in response to TGF-β1 over the same period of time. These results demonstrate that TGF-β1 effects on miR-1207-5p expression are not occurring through transcriptional up-regulation, and instead suggest that the changes result from post-transcriptional modulation. Davis et al. [17], [18] reported that the signal transducers of TGF-β/bone morphogenic proteins (BMP), the Smads, modulate miRNA expression post-transcriptionally through pri-miRNA binding and regulation of miRNA-processing. Only miRNAs containing the RNA Smad binding element (R-SBE) in the stem region are regulated by TGF-β/BMP. Pri-miR-1207 does not contain the main consensus sequence for Smad binding (5′-CAGAC-3′) in the stem region, but it has two copies of a variant of this sequence (5′-CAGGG-3′), which has been also found in miRNAs regulated post-transcriptionally by TGF- β/BMP [18].

**Figure 8:**
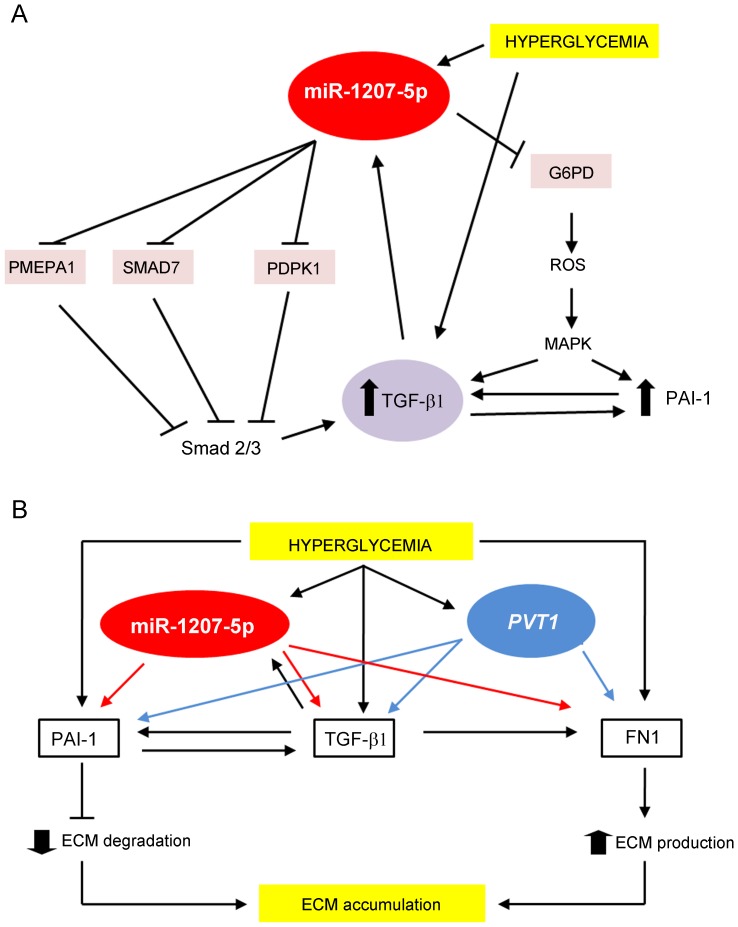
Potential mechanisms for miR-1207-5p and *PVT1* contribution to ECM accumulation in the kidney under hyperglycemic conditions. (A) Hyperglycemia increases miR-1207-5p level, which downregulates *G6PD*, *PMEPA1*, *PDPK1* and *SMAD7*. G6PD (glucose-6-phosphate dehydrogenase) is the first and rate-limiting enzyme of the pentose pathway, which results in the production of NADPH and ribose-5-phosphate. PMEPA1 (prostate transmembrane protein, androgen induced 1) antagonizes TGF-β signaling by sequestering phosphorylated Smad2 and Smad3 to prevent their nuclear translocation [45]. PMEPA1 negatively impact on the duration and intensity of TGF- β/Smad signaling. PDPK1 (3-phosphoinositide-dependent protein kinase-1) negatively regulates TGF-β signaling by preventing translocation of Smad3 and Smad4 from the cytoplasm to the nucleus, and Smad7 from the nucleus to the cytoplasm [47]. Smad7 antagonizes TGF-β family by forming a stable complex with TGF-β type I receptor and, therefore, leading to inhibition of Smad2/3 phosphorylation [57]. Direct downregulation of G6PD by miR-1207-5p may induce increased production of reactive oxygen species (ROS). Increased ROS up-regulates proteins involved in ECM accumulation in the kidney, including TGF-β and PAI-1 (B) Proposed mechanism for a joint and independent effect of miR-1207-5p and *PVT1* on ECM accumulation under hyperglycemic conditions. PAI-1, plasminogen-activator inhibitor 1; TGF-β, transforming growth factor beta; FN1, fibronectin 1; ECM, extracellular matrix.

Among the many potential target genes for miR-1207-5p, we identified four candidates based on known relationships with TGF-β and PAI-1, which play important roles in the regulation of ECM accumulation in the kidney (Fig. 8A). These include glucose-6-phosphate dehydrogenase (G6PD), which is the first and rate-limiting enzyme of the pentose pathway that produces NADPH, the principal intracellular antioxidant for all cells. Direct down-regulation of G6PD by miR-1207-5p, as we show here, may lead to lower NADPH levels, which in turn, may induce increased production of reactive oxygen species (ROS). Increased ROS up-regulates proteins involved in ECM accumulation in the kidney, including TGF-β and PAI-1 [43], [44]. In addition, prostate transmembrane protein, androgen induced 1 (PMEPA1) antagonizes TGF-β signaling by sequestering phosphorylated Smad2 and Smad3 to prevent their nuclear translocation [45]. Thus, the direct down-regulation of PMEPA1 by miR-1207-5p may increase duration and intensity of TGF-β activity [12], [46]. The third candidate, 3-phosphoinositide-dependent protein kinase-1 (PDPK1), negatively regulates TGF-β signaling by preventing translocation of Smad3 and Smad4 from the cytoplasm to the nucleus. In agreement with these results, we found that phosphorylated Smad3 (p-Smad3) increased in cells with miR-1207-5p over-expression, and decreased in cells with miR-1207-5p knockdown compared to controls, suggesting that the augmented TGF-β activity is at least partially mediated by a Smad3 (Fig. 8A). Other mechanism by which PDPK1 negatively regulates TGF-β signaling is by preventing Smad7 translocation from the nucleus to the cytoplasm [47], [48]. Smad7 plays a protective role in diabetic renal injury by blocking the activation of both TGF-β/Smad and nuclear factor-κB (NF- κB) signaling pathways [49], [50], We found that miR-1207-5p directly targets and decreases the expression of both PDPK1 and Smad7, which may together contribute to increased TGF-β activity and ECM accumulation (Fig. 8A).

In agreement with the observed down-regulation of *G6PD*, *PMEPA1*, *PDPK1*, and *SMAD7* by miR-1207-5p, and the known effect of these target genes on TGF-β expression and activity, we found that levels of TGF-β1, PAI-1 and FN1 mRNA and secreted protein increased in MC over-expressing miR-1207-5p compared to control cells. TGF-β1 is an effective inducer of PAI-1 expression because, following nuclear translocation, the Smad2/3-Co-Smad4 complex binds to the PAI-1 promoter and induces its expression [1]. TGF-β1 also increases FN1 accumulation, a major component of ECM [1]. We found that *PVT1* over-expression increased TGF-β1, PAI-1 and FN1 mRNA and secreted protein in MC similar to what was observed in cells over-expressing miR-1207-5p. Increased expression of *PVT1* corresponded to higher levels of *FN1* mRNA, while conversely, *PVT1* knockdown correlated with reduced *FN1* expression. MC treated with SB431542, a potent inhibitor of TGF-β type I receptor that specifically decreases TGF-β signaling [23], showed a reduction in *FN1* mRNA, which decreased even more with *PVT1* knockdown. These results suggest that part of the effect of *PVT1* on the expression of *FN1* is independent of TGF-β.

The results presented here demonstrate that an increase in miR-1207-5p under hyperglycemic conditions contributes to ECM accumulation in the kidney. The dysregulation of miRNA levels that might affect different tissues and organs in a particular disease is one of the three main mechanisms by which miRNAs can be involved in disease pathogenesis. The second mechanism is less frequent and involves a genetic variation that alters the biogenesis and/or binding of that miRNA to a causal gene. In a third mechanism, a miRNA associated single nucleotide polymorphisms (miRSNPs) found on miRNA target sites within 3′UTRs can eliminate or weaken the binding of a miRNA to its target site or increase the binding by creating a perfect sequence match to the seed of a miRNA that normally is not associated with the given mRNA. For example, a miRNSNP C1936T (rs13385) was recently described on a miR-1207-5p target site within the 3′UTR of the Heparin binding epidermal growth factor (*HBEGF*) gene [51]. HBEGF is a growth factor highly expressed in podocytes, tubular epithelial cells, and mesangial cells [52]. When undifferentiated podocytes were transfected with miR-1207-5p mimics, the HBEGF protein levels were reduced by about 50%. However, the presence of 1936T variant prevented miR-1207-5p down-regulation of HBEGF in podocytes, thus resulting in higher HBEGF protein levels [51]. Interestingly, the inheritance of the miRSNP 1936T allele was significantly associated to chronic renal failure in a cohort of 78 patients diagnosed with complement factor H-related protein 5 (CFHR5) nephropathy (also known as C3-glomerulopathy). Thus, miR-1207-5p seems to have a protective role in CFHR5 nephropathy when, in the presence of the 1936C allele, is able to bind to the 3′UTR of HBEGF and down-regulate its expression [51]. High expression of HBEGF in kidney cells have been associated with increased mesangial and ephitelial cell proliferation as well as renal fibrosis [53]. However, the exact mechanism by which HBEGF can alter disease phenotype in CFHR5 nephropathy is currently unknown. At any rate, miR-1207-5p seems to have a protective role in the non-diabetic immune CFHR5 nephropathy as reported by Papagregoriou et al. [51], in contrast with the results that we are presenting here that associate this miRNA with diabetic nephropathy. These apparent contradictory findings may be explained by the different type of kidney cells (mesangial cells vs. podocytes) used in our study compared to Papagregoriou et al. [51] as well as the different etiology and pathophysiology of diabetic nephropathy compared to CFHR5 nephropathy.

The discovery that inhibition of miRNA expression *in vivo* is feasible, as well as the recent results from successful clinical trials using this technology, opens the way for future novel therapeutic applications, including the inhibition of those miRNAs that are commonly up-regulated in diabetic nephropathy [8], [54]. Specifically, we found that the level of secreted TGF-β1, PAI-1 and FN1 protein decreases in MC with miR-1207-5p knockdown. These results suggest that the developing a therapeutic against miR-1207-5p may help to prevent diabetic nephropathy and/or slow its progression. Although miR-1207-5p and its host gene act independently from each other, we hypothesize that they both contribute to the excessive ECM accumulation in the kidney under hyperglycemic conditions, a hallmark of diabetic nephropathy (Fig. 8B). Further characterization of miR-1207-5p and its host gene, the long non-coding RNA *PVT1,* may yield new insights into the complex pathogenesis of diabetic nephropathy, and may contribute to the development of new and more effective treatments based on these new potential therapeutic targets.

## Methods

### Cell culture

Primary cultures of MC and RPTEC were purchased from Lonza (Walkersville, MA). MC were cultured in Lonza Mesangial Cell Basal Medium (MsBM) supplemented with 5% fetal bovine serum (FBS), and RPTEC were placed in renal epithelial cell basal medium and growth supplements, according to the manufacturer's instructions. Briefly, approximately 3500 MC/cm^2^ or 2500 RPTEC/cm^2^ were seeded in 25 cm^2^ cell culture flasks (Corning Life Sciences; Lowell, MA) containing 5 ml of cell culture medium. Cells were placed at 37°C in a Hera Cell 5% CO_2_ incubator (ThermoFisher Scientific; Waltham, MA). Culture medium was replaced the first day after seeding and then again every two days. Cells were subcultured every 7 to 9 days using Clonectis ReagentPack Subculture Reagents (Lonza) according to the manufacturer's instructions. All experiments were performed using cells between the sixth and eighth passage.

A conditionally immortalized human podocyte cell line was kindly shared by Dr. Moin Saleem (Bristol Royal Hospital for Children; Bristol, UK) [55]. Podocytes were cultured in RPMI-1640 (Sigma; St Louis, MO) supplemented with 10 µg/ml insulin, 5.5 µg/ml transferrin, 5 ng/ml selenium, and 10% FBS (Sigma) according to Saleem et al. [55]. Cells were incubated at 33°C until they reached 50-60% confluency, after which the temperature was increased to 38°C. Culture medium was replaced every two days, and cells were fully differentiated and harvested after 14 days of culturing at 38°C.

### Cell treatments

Approximately 90,000 MC were seeded per 25 cm^2^ flask in 5 ml MsBM supplemented with 5% FBS, and placed overnight in a 37°C incubator. Cells were serum-starved overnight just prior to initiating treatments with normal glucose (NG: 5.6 mM), high glucose (HG: 30 mM) or osmolarity control (OC: 5.6 mM glucose +24.4 mM mannitol) for 48 h.

For the TGF-β1 dose-response experiments, 80,000 MC/well were seeded in a 12-well plate containing 1 ml MsBM and 5% FBS. MC were serum-starved overnight and then treated for 24 h with serum-free medium supplemented with 2.5, 5.0, or 10.0 ng/ml TGF-β1 (R&D Systems; Minneapolis, MN). For the TGF-β1 time-course experiments, MC were incubated for 10, 20, 30, 60 min, 6 h, or 24 h in media containing 10 ng/ml TGF-β1.

### Bioinformatics

Predicted miRNA gene targets were identified using the TargetScan software, release June 2012 (http://www.targetscan.org) [19]–[21]. Candidate miR-1207-5p targets predicted to contribute to a decrease in TGF-β, PAI-I, FN1, and COL4A1 were identified using the Pathway Studio software (Elsevier; Rockville, MD) [22].

### Transfection of miR-1207-5p mimics and anti-miR-1207-5p inhibitors

Approximately 70,000 MC/well were seeded in a 12-well plate and incubated overnight at 37°C. Cells were transfected with 3 µl Lipofectamine RNAiMAX (Life Technologies; Carlsbad, CA) mixed with 30 nM meridian miR-1207-5p or negative control mimic, and 50 nM anti-miR-1207-5p or negative control (Dharmacon, Thermo Fisher Scientific; Pittsburgh PA). Total RNA was extracted 48 h after transfection using the miRNeasy Kit (Qiagen; Valencia, CA) according to the manufacturer's instructions. Verification of miR-1207-5p over-expression and knockdown was determined using qPCR, as described below.

### 3′ UTR Luciferase Reporter Assays

Reporters were constructed based on pmirGLO Dual Luciferase miRNA Target Expression Vector (Promega; Fitchburg, WI). The pmirGLO vector is based on Promega dual-luciferase technology, with firefly luciferase used as the primary reporter to monitor mRNA regulation and Renilla luciferase acting as a control reporter for normalization.

The 3′UTR sequence of human *G6PD* (∼500), *PMEPA1* (∼700 bp), *SMAD7* (∼730) and *PDPK1* (∼4.8 kb) were generated by PCR using human genomic DNA as template and the CloneAmp HiFi PCR kit (Clontech; Mountain View, CA) as per the manufacturer's instructions. Restriction sites for PmeI and XbaI as well as 15 bp homologous to the vector sequence were added to the PCR primers (sequences provided in Table S1) to allow the cloning of the PCR products into the MCR of the pmirGLO vector using the Cold Fusion Cloning kit (System Biosciences; Mountain View, CA). All the reporter plasmids were sequenced to make sure that the predicted miR-1207-5p sites were preserved and no additional sites were introduced by the cloning.

About 20,000 human embryonic kidney 293 (HEK293) cells were seeded per well into a white 96-well plate. After overnight incubation at 37°C, cells were co-transfected with 100 ng of the indicated 3′UTR luciferase reporter vectors and 30 nM miR-1207-5p or negative control mimic (Dharmacon) using 0.2 µl per well of Lipofectamine 2000 (Life Technologies). HEK293 cells were also co-transfected with reporter vectors and 50 nM miR-1207-5p inhibitor or negative control (Dharmacon). Luciferase activity was measured 48 h after transfection using the Dual-Glo Luciferase Assay System (Promega). Firefly luciferase activity was normalized to the corresponding renilla luciferase activity and plotted as a percentage of the control (HEK293 cells co-transfected with plasmid and control mimic or inhibitor). Experiments were performed in quadruplicate wells of 96-well plate.

### PVT1 over-expression in MC

MC were transfected with plasmid pCMV-PVT1 or empty vector pCMV (Origene; Rockville, MD) by electroporation using the Neon System (Life Technologies). About 400,000 MC were mixed with 30 µg pCMV-PVT1 or empty vector just before electroporating the cells, according to the manufacturer's instructions. We found that the best conditions for MC electroporation were those established using program 14 in the Neon System (pulse, 1200 V; pulse width, 20; number of pulses, 2). Immediately after transfection, cells were seeded in a 6-well plate containing MsBM supplemented with 5% FBS and incubated at 37°C for 48 h. Total RNA was then extracted, reverse transcribed, and quantified by qPCR.

### PVT1 knockdown using small interfering RNA (siRNA) in MC

Two siRNAs targeting different parts of *PVT1* were used in this study: PVT1a siRNA targets exon 2 [56] and PVT1-tv6 siRNA [11] (Fig. 1A), designed from accession number BG110543 using Dharmacon siDesign (www.dharmacon.com). The target sequence of siPVT1a was 5-CAGCCATCATGATGGTACT-3 and for PVT1-tv6 siRNA was 5′-GCATGGACTTGCAGGCCAA-3′. The EST BG110543 contains marker rs13447075, which was previously found to be associated with ESRD [10]. Approximately 2×10^5^ MC were plated per 25 cm^2^ flask at least 24 h before transfection to achieve 50–70% confluency, then transfected with either 30 nM PVT1a siRNA, PVT1-tv6 siRNA, or negative control siRNA comprised of sequence not found in the human genome (Life Technologies) using 5 µl Lipofectamine RNAiMax (Life Technologies) per ml of MsBM media, following the manufacturer's instructions. Cell transfections were done in the presence or absence of 1 µM SB431542, a potent and specific inhibitor of TGF-β type I receptor [23]. Cells and cell culture media were harvested 48 h post-transfection for RNA and protein analysis.

### Total RNA extraction and quantification

Total RNA was extracted using the miRNeasy Mini Kit (Qiagen) according to the manufacturer's protocol. RNA concentration was determined by absorbance at 260 nm and RNA integrity was evaluated using the RNA 6000 Nano Lap Chip Kit; only RNA samples with a RIN >8 and 18S/28S ratio >2.0 were used in the qPCR assays.

### Quantitative real-time RT-PCR (qPCR)

First-strand cDNA was synthesized from total RNA obtained from MC, RPTEC, podocytes, or the Human Total RNA Survey Panel (Life Technologies) using random primers and the Super Script III Reverse Transcriptase kit (Life Technologies) according to the manufacturer's protocol. For the quantification of specific RNAs, including pri-miR-1207, total RNA samples were reverse-transcribed using the TaqMan MicroRNA Reverse Transcriptase kit (Life Technologies), as per the manufacturer's instructions. Quantitative real time RT-PCR (qPCR) was performed using commercial TaqMan Gene Expression Assays (Life Technologies) in conjunction with the ABI Prism 7900 HT Sequence Detector apparatus (Life Technologies). Data were normalized using both *PPIA* (cyclophilin A) and *UBC* (ubiquitin C), the two most stable housekeeping genes tested for the experimental conditions used. For mature miRNA quantification, data were normalized using RNU6B and RNU44. Results were analyzed with RQ Manager and DataAssist software (Life Technologies). Results were analyzed with RQ Manager software (Life Technologies), and qBasePlus v1.5 (Biogazelle NV; Ghent, Belgium). Assay information is provided in the Table S2.

### Protein extraction and quantification

Cell culture media from MC was treated with 0.5 ml absolute ethanol/ ml of media for a final 33% v/v ethanol and stored at -20°C for at least 2 hours to precipitate proteins. Samples were then centrifuged at 4,000× *g* for 30 min at 4°C, and the protein precipitate was resuspended in 400 µl of PBS supplemented with Complete Protease Inhibitor Cocktail (Roche; Indianapolis, IN). Total protein content for each sample was determined using the BCA Protein Assay kit (Pierce; Rockford, IL) according to the manufacturer's instructions.

### Enzyme-linked immunosorbent assay analysis (ELISA)

Secreted TGF-β1, FN1 and PAI-1 protein in cell culture media was quantified using a commercial sandwich ELISA kit for TGF-β1 (R&D Systems), FN1 and PAI-1 (eBioscience; San Diego, CA), according to the manufacturer's instructions. The reaction was stopped after 5 min with 1N H_2_SO_4_, and the optical density was read at 450 nm using a VICTOR_3_ Microplate Reader (Perkin Elmer; Waltham, MA). Results were normalized by content of total soluble protein quantified using the BCA Protein Assay kit (Pierce).

Activated SMAD3 was measured using phospho-SMAD3 (ser423/425) Instant One ELISA (eBiosciences). MCs were transfected with mir-1207-5p mimics and inhibitors as we previously described in this section. About 24 h after transfection, cells were stimulated with high glucose [30 mM] and incubated for another 24 h. Afterwards, cell culture media was removed, and cells were lysed in 100 µl per well of 1× Cell Lysis Mix (ELISA kit component) by incubation for 10 min at room temperature with shaking (300 rpm). Total soluble protein was quantified by BCA Protein Assay kit and the same amount of protein was loaded per well (about 1 µg/well). Fifty microliters of antibody cocktail (capture antibody + detection antibody reagents from the ELISA kit) were added per well and incubated for 1 h at room temperature. After three washes with 200 µl 1 × Wash Buffer per well, 100 µl of Detection Reagent (kit component) were added per well and incubated for 30 min at room temperature. Finally, the reaction was interrupted by adding 100 µl of Stop Solution (kit component) and the plate was read by measuring absorbance of the samples using a colorimetric plate reader at 450 nm.

### Statistical analysis

All statistical analyses were performed using the software Graph Pad Prism 5 for Microsoft Windows. One-way ANOVA test with a Dunnett's Multiple Comparison post-test were used to assess differences between conditions. Results were considered statistically significant if *P*<0.05.

## Supporting Information

Table S1 **Primers used for the cloning of luciferase reporter vectors.**
(DOC)

Table S2 **TaqMan assays used in real-time quantitative PCR (qPCR).**
(DOC)
