# Correction: Role of MicroRNA 1207-5P and Its Host Gene, the Long Non-Coding RNA *Pvt1*, as Mediators of Extracellular Matrix Accumulation in the Kidney: Implications for Diabetic Nephropathy

**DOI:** 10.1371/journal.pone.0168353

**Published:** 2016-12-09

**Authors:** M. Lucrecia Alvarez, Mahdieh Khosroheidari, Elena Eddy, Jeff Kiefer, Johanna K. DiStefano

The author list for this article is corrected to list Dr. Johanna K. DiStefano as the fifth author of the article. Johanna DiStefano’s inclusion as an author is in line with the recommendation of the Translational Genomics Research Institute, which evaluated the contributions to this work.

The first and corresponding author M. Lucrecia Alvarez disagrees with the addition of Johanna K. DiStefano to the author list, as she considers that Dr. DiStefano did not contribute to this publication. Dr. Alvarez also states her disagreement to the additions to the Author Contributions listed below.

The revised author list is: M. Lucrecia Alvarez, Mahdieh Khosroheidari, Elena Eddy, Jeff Kiefer, Johanna K. DiStefano

The affiliation for Johanna DiStefano at the time of the study was: Diabetes, Cardiovascular and Metabolic Diseases Center, Translational Genomics Research Institute, Phoenix

The revised Authors Contributions are: Conceived and designed the experiments: MLA JKD. Performed the experiments: MLA MK EE. Analyzed the data: MLA MK EE JK. Contributed reagents/materials/analysis tools: MLA. Wrote the paper: MLA JKD.

The revised citation is: Alvarez ML, Khosroheidari M, Eddy E, Kiefer J, DiStefano JK (2013) Role of MicroRNA 1207-5P and Its Host Gene, the Long Non-Coding RNA *Pvt1*, as Mediators of Extracellular Matrix Accumulation in the Kidney: Implications for Diabetic Nephropathy. PLoS ONE 8(10): e77468. doi:10.1371/journal.pone.0077468
