# Supplementary material for: Role of MicroRNA 1207-5P and Its Host Gene, the Long Non-Coding RNA Pvt1, as Mediators of Extracellular Matrix Accumulation in the Kidney: Implications for Diabetic Nephropathy
Source: PLoS One. 2013 Oct 25;8(10):e77468. doi: 10.1371/journal.pone.0077468 (PMC3808414; doi:10.1371/journal.pone.0077468)
Supplement: Table S1 — Primers used for the cloning of luciferase reporter vectors. (DOC) [file pone.0077468.s001.doc]

**Table S1**: Primers used for the cloning of luciferase reporter vectors

| **3'UTR** | **Sense primer** | **Antisense primer** | **PCR product size [bp]** |
| --- | --- | --- | --- |
| G6PD | 5’GTGTAATTCTAGTTGTTTAAACTCGCTGCTGCTACTACCC 3’ | 5’TGCCTGCAGGTCGACTCTAGAATGGCAGGGCATTGAGGT 3’ | 510 |
| PMEPA1 | 5’GTGTAATTCTAGTTGTTTAAATAAGCTCTCGGCTTCTCTGC 3’ | 5’TGCCTGCAGGTCGACTCTAGACTGCAAAGGAGAGCAGTTCC 3’ | 750 |
| SMAD7 | 5’GTGTAATTCTAGTTGTTTAAAAGGTATGATCGGCAGGACAC 3’ | 5’TGCCTGCAGGTCGACTCTAGATGGGTTATGACGGACCAAAT 3’ | 780 |
| PDPK1 | 5’GTGTAATTCTAGTTGTTTAAATTCGCTGCCAGGACACCT 3’ | 5’TGCCTGCAGGTCGACTCTAGATGCCAGGTGGTTAGGCTATG 3’ | 4,800 |
