# Supplementary material for: Role of MicroRNA 1207-5P and Its Host Gene, the Long Non-Coding RNA Pvt1, as Mediators of Extracellular Matrix Accumulation in the Kidney: Implications for Diabetic Nephropathy
Source: PLoS One. 2013 Oct 25;8(10):e77468. doi: 10.1371/journal.pone.0077468 (PMC3808414; doi:10.1371/journal.pone.0077468)
Supplement: Table S2 — TaqMan assays used in real-time quantitative PCR (qPCR). (DOC) [file pone.0077468.s002.doc]

**Table S2**: TaqMan assays used in real-time quantitative PCR (qPCR)

| **Gene symbol** | **Gene name** | **TaqMan Assay (*)** | | **Ref Seq** | | **Assay Location** | | **Exon boundary** | | **Amplicon length (bp)** | |
| --- | --- | --- | --- | --- | --- | --- | --- | --- | --- | --- | --- |
| *UBC* | Ubiquitin C | Hs00824723_m1 | NM_021009.4 | | 445 | | 1-2 | | 71 | |  |
| 18S | Eukaryotic 18S ribosomal RNA | Hs99999901_s1 | X03205.1 | | 604 | | 1-1 | | 187 | |  |
| *PPIA* | Peptidylprolyl isomerase A (cyclophilin A) | Hs99999904_m1 | NM_021130.3 | | 436 | | 4-4 | | 98 | |  |
| *PVT1* | Pvt1 oncogene (non-protein coding) | Hs01069044_m1 | NR_003367.1 | | 1374 | | 6-7 | | 83 | |  |
| *FN1* | Fibronectin 1 | Hs01549976_m1 | Multiple | | 1482 | | 8-9 | | 81 | |  |
| *SERPINE1* | Serpin peptidase inhibitor, member 1 or plasminogen activator inhibitor, type 1 | Hs00167155_m1 | NM_001165413.1 | | 371 | | 3-4 | | 82 | |  |
| *TGFB1* | Transforming growth factor, beta 1 | Hs99999918_m1 | NM_00660.4 | | 1598 | | 4-5 | | 125 | |  |
| *G6PD* | Glucose-6-phosphate dehydrogenase | Hs00166169_m1 | NM_000402.3  NM_001042351.1 | | 361 | | 2-3 | | 94 | |  |
| *PMEPA1* | Prostate transmembrane protein, androgen induced 1 | Hs00375306_m1 | multiple | | multiple | | 2-3 | | 77 | |  |
| *PDPK1* | 3-phosphoinositide dependent protein kinase-1 | Hs00176884_m1 | multiple | | multiple | | 11-12 | | 70 | |  |
| *SMAD7* | SMAD family member 7 | Hs00998193_m1 | multiple | | multiple | | 3-4 | | 105 | |  |
| *hsa-pri-miR-1207* | Primary miR-1207 | Hs03305481_pri | MIPF0000596 | |  | |  | | 62 | |  |
| *RNU6B* |  | 001002 | NR_002444 | |  | |  | |  | |  |
| *RNU44* |  | 001094 | NR_002750 | |  | |  | |  | |  |
| *hsa-miR-1204* |  | 002872 | MIMAT0005868 | |  | |  | |  | |  |
| *hsa-miR-1205* |  | 002778 | MIMAT0005869 | |  | |  | |  | |  |
| *hsa-miR-1206* |  | 002878 | MIMAT0005870 | |  | |  | |  | |  |
| *has-miR-1207-3p* |  | 002826 | MIMAT0005872 | |  | |  | |  | |  |
| *hsa-miR-1207-5p* |  | 241060_mat | MIMAT0005871 | |  | |  | |  | |  |
| *hsa-miR-1208* |  | 002880 | MIMAT0005873 | |  | |  | |  | |  |

(*) TaqMan Assay: inventoried pre-designed TaqMan Gene Expression Assays (Life Technologies; Foster City, CA). Sequences of primers and probe are proprietary of Life Technologies.
